# Correction to: The microbiome as a biosensor: functional profiles elucidate hidden stress in hosts

**DOI:** 10.1186/s40168-020-00883-0

**Published:** 2020-06-30

**Authors:** Avihai Zolti, Stefan J. Green, Noa Sela, Yitzhak Hadar, Dror Minz

**Affiliations:** 1 Department of Plant Pathology and Microbiology, Robert H. Smith Faculty of Agriculture, Food and Environment, The Hebrew University of Jerusalem, 76100 Rehovot, Israel; 2 Institute of Soil, Water and Environmental Sciences, Agricultural Research Organization–Volcani Center, 7528809 Rishon Lezion, Israel; 3 Sequencing Core, Research Resources Center, University of Illinois at Chicago, Chicago, IL USA

**Correction to: Microbiome 8, 71 (2020)**

**https://doi.org/10.1186/s40168-020-00850-9**

Following publication of the original article [1], an error was identified in the Fig. 7 legend.

The updated change has been highlighted in bold typeface.

The legend currently reads:

“Conceptual model of hypothetical bacteria harboring physiological features (i.e., genes, pathways and modules) associated with water quality. Features enriched in **a TWW- or b FW**-irrigated root microbiomes. White symbols indicate features that are significantly enriched at the DNA level (metagenomes), grey features are highly expressed (metatranscriptomes), and green features are significantly abundant and expressed in one treatment relative to the other”.

The legend should read:

“Conceptual model of hypothetical bacteria harboring physiological features (i.e., genes, pathways and modules) associated with water quality. Features enriched in **a FW- or b TWW**-irrigated root microbiomes. White symbols indicate features that are significantly enriched at the DNA level (metagenomes), grey features are highly expressed (metatranscriptomes), and green features are significantly abundant and expressed in one treatment relative to the other”.
