# Supplementary material for: The microbiome as a biosensor: functional profiles elucidate hidden stress in hosts
Source: Microbiome. 2020 May 21;8:71. doi: 10.1186/s40168-020-00850-9 (PMC7243336; doi:10.1186/s40168-020-00850-9)
Supplement: Supplementary file 2 — Additional file 1: Figure S1. Non-metric multidimensional scaling (nMDS) based on the Bray-Curtis dissimilarity index of the SEED annotated gene counts for (a) metagenome, and (b) metatranscriptome. Figure S2. Correlation between metagenomics enriched SEED categories and soil environmental parameters, for (a) subsystems enriched in TWW irrigated roots, or (b) enriched in FW irrigated roots. Figure S3. Metagenomic enriched KEGG modules in (a) FW irrigated roots, or (b) TWW irrigated roots. The bar chart present the log10(P value) of the enrichment analysis calculated by KeggProfiler ‘R’ package. Figure S4. Metatransctiptome enrichement KEGG modules in (a) FW irrigated roots, or (b) TWW irrigated roots. The bar chart present the log10(P value) of the enrichment analysis calculated by KeggProfiler ‘R’ package. Figure S5. Microbial gene expression patterns revealed TWW irrigation enrichment of arginine and proline metabolism genes, highlighting those in the arginine-to-spermidine pathway (colored in pink). Figure S6. Meta- analysis of the full nqr operon in environmental metagenomes. Box plot demonstrates the nqr operon (a-f subunits) gene abundance in acidic pH (<7), neutral (7-8), or alkaline (>8) pH. Figure S7. Meta- analysis of the full mnh operon in environmental metagenomes. Box plot demonstrates the mnh operon (a-g subunits) gene abundance in acidic pH (<7), neutral (7-8), or alkaline (>8) pH. [file 40168_2020_850_MOESM1_ESM.pptx]

## Slide 1
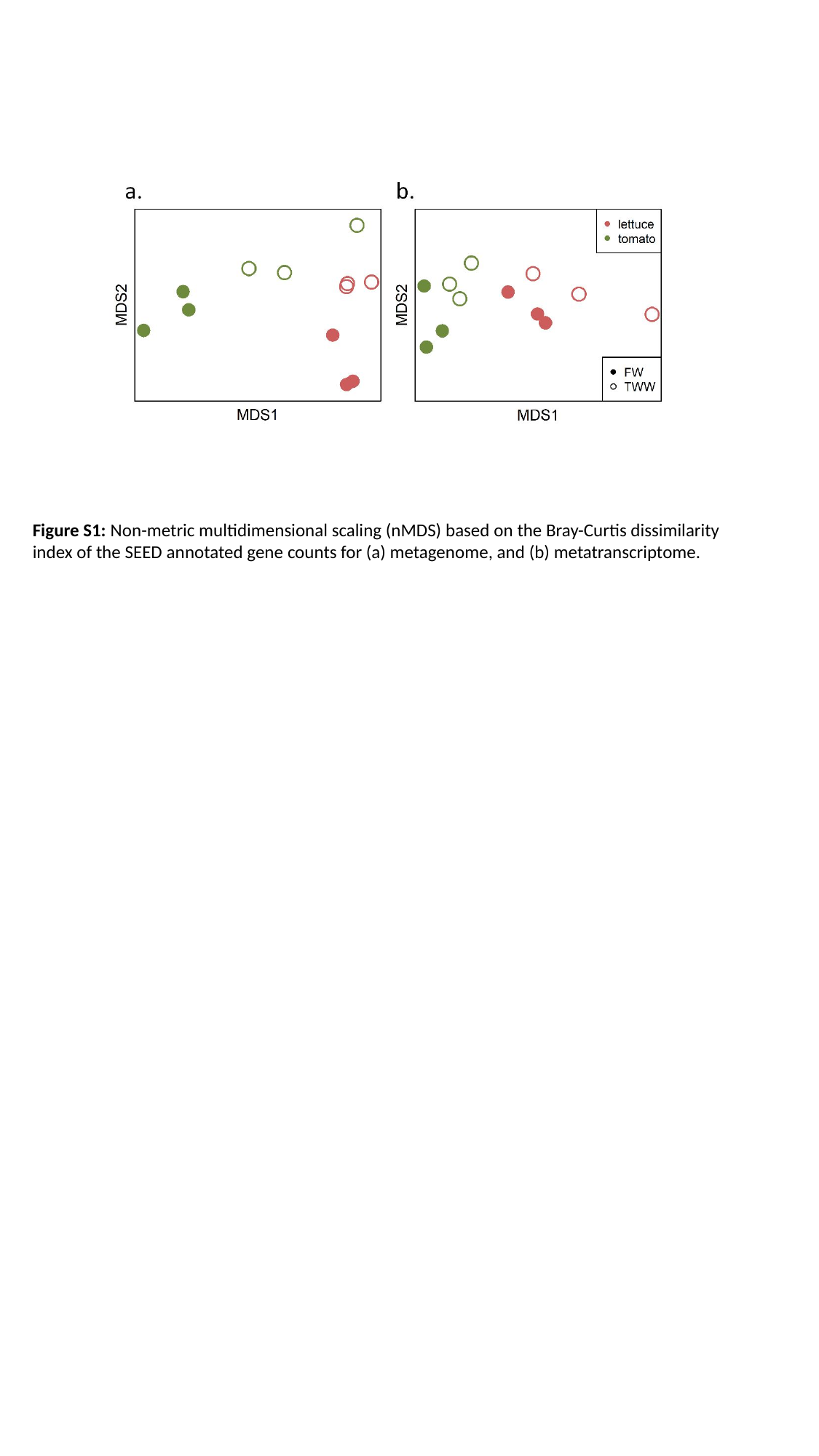

Figure S1: Non-metric multidimensional scaling (nMDS) based on the Bray-Curtis dissimilarity index of the SEED annotated gene counts for (a) metagenome, and (b) metatranscriptome.

## Slide 2
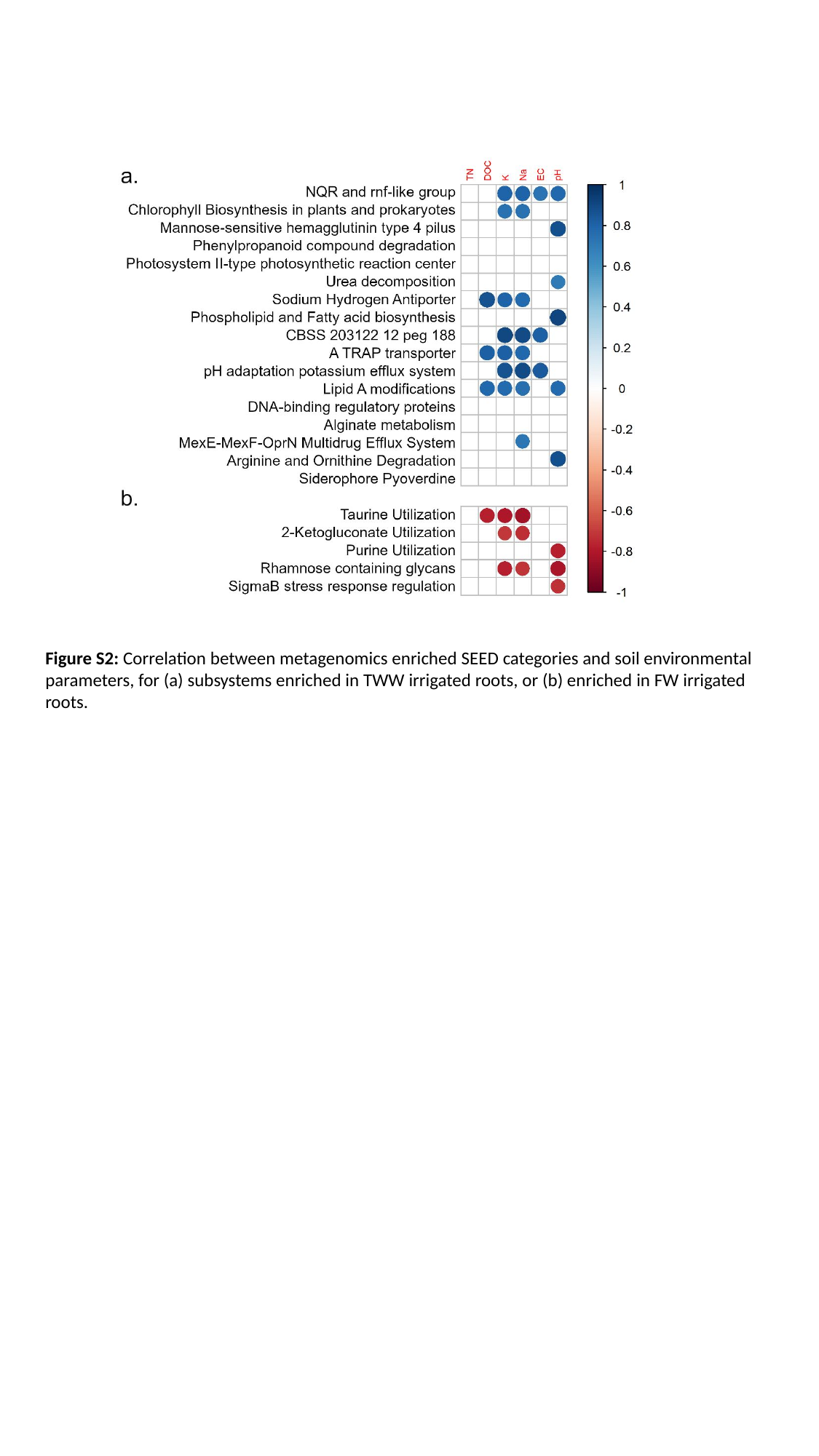

Figure S2: Correlation between metagenomics enriched SEED categories and soil environmental parameters, for (a) subsystems enriched in TWW irrigated roots, or (b) enriched in FW irrigated roots.

## Slide 3
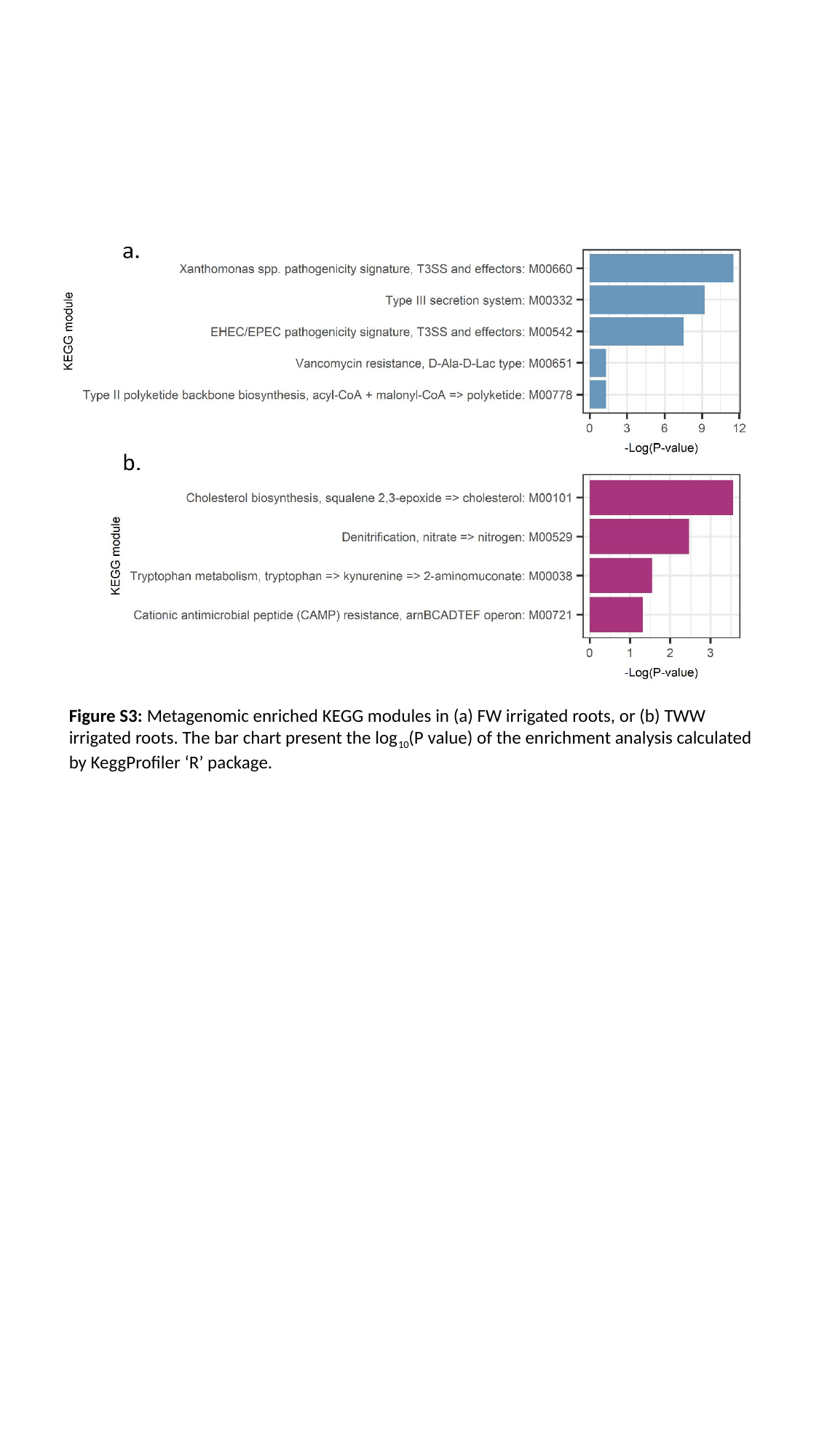

Figure S3: Metagenomic enriched KEGG modules in (a) FW irrigated roots, or (b) TWW irrigated roots. The bar chart present the log10(P value) of the enrichment analysis calculated by KeggProfiler ‘R’ package.

## Slide 4
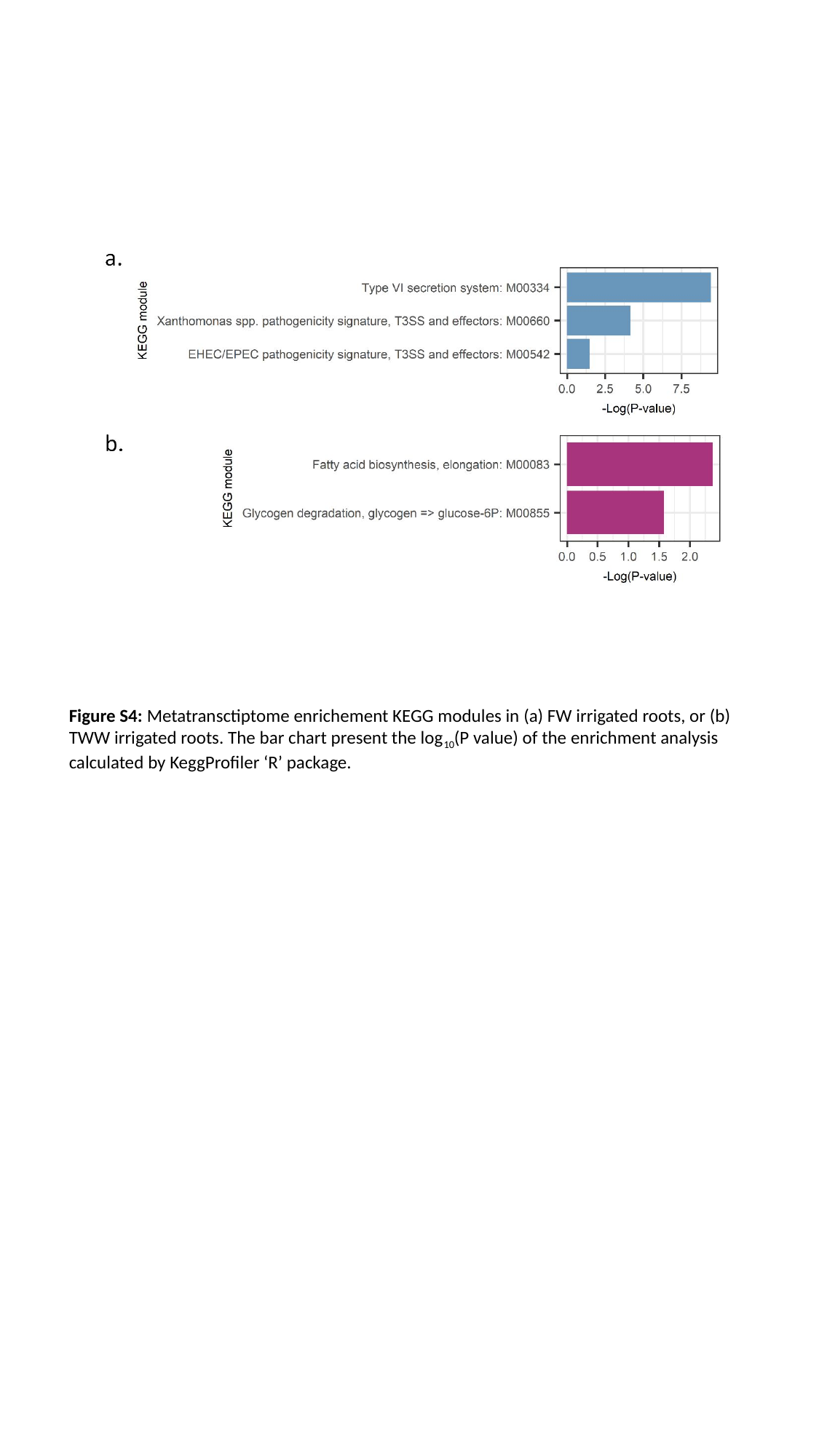

Figure S4: Metatransctiptome enrichement KEGG modules in (a) FW irrigated roots, or (b) TWW irrigated roots. The bar chart present the log10(P value) of the enrichment analysis calculated by KeggProfiler ‘R’ package.

## Slide 5
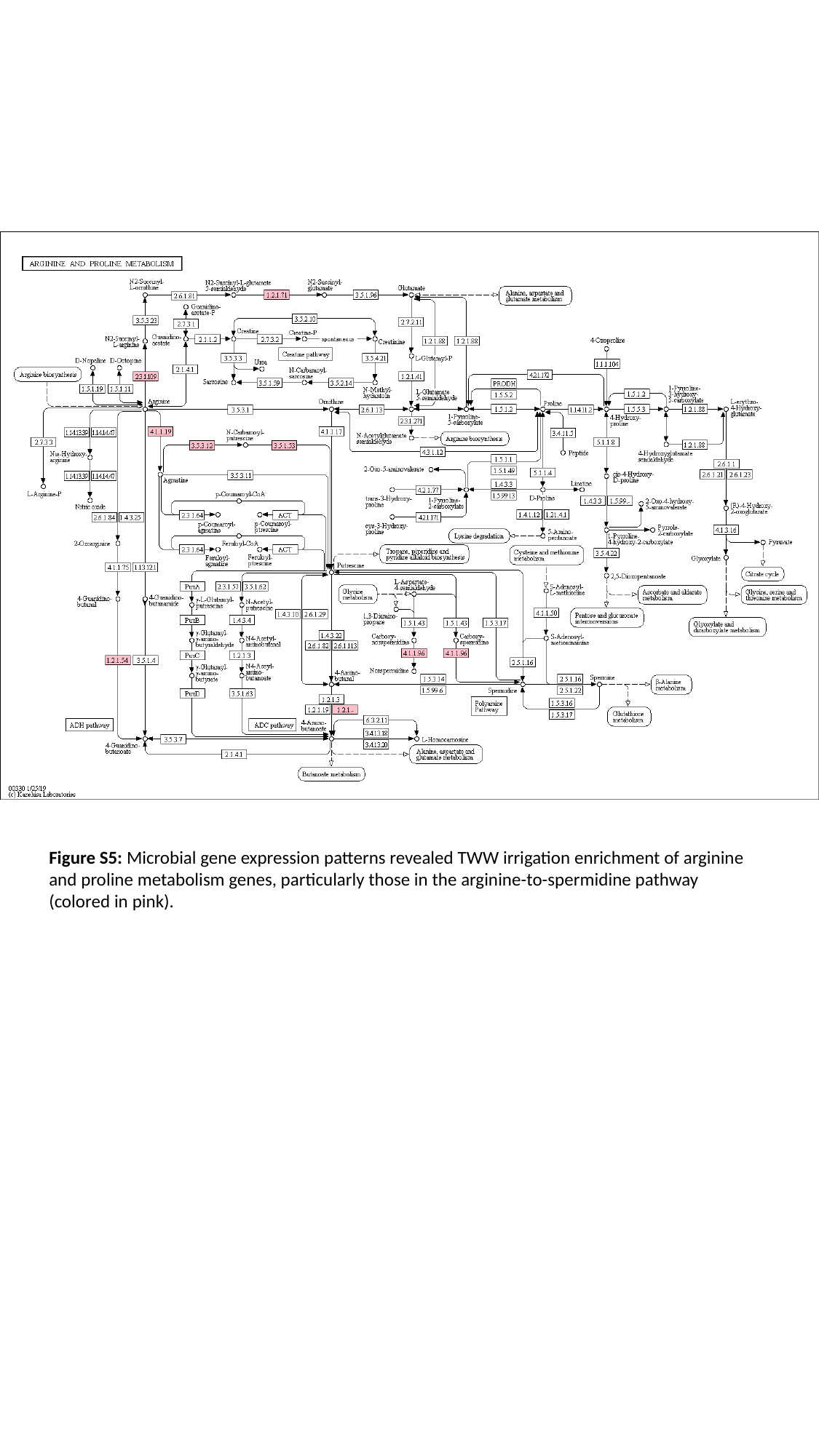

Figure S5: Microbial gene expression patterns revealed TWW irrigation enrichment of arginine and proline metabolism genes, particularly those in the arginine-to-spermidine pathway (colored in pink).

## Slide 6
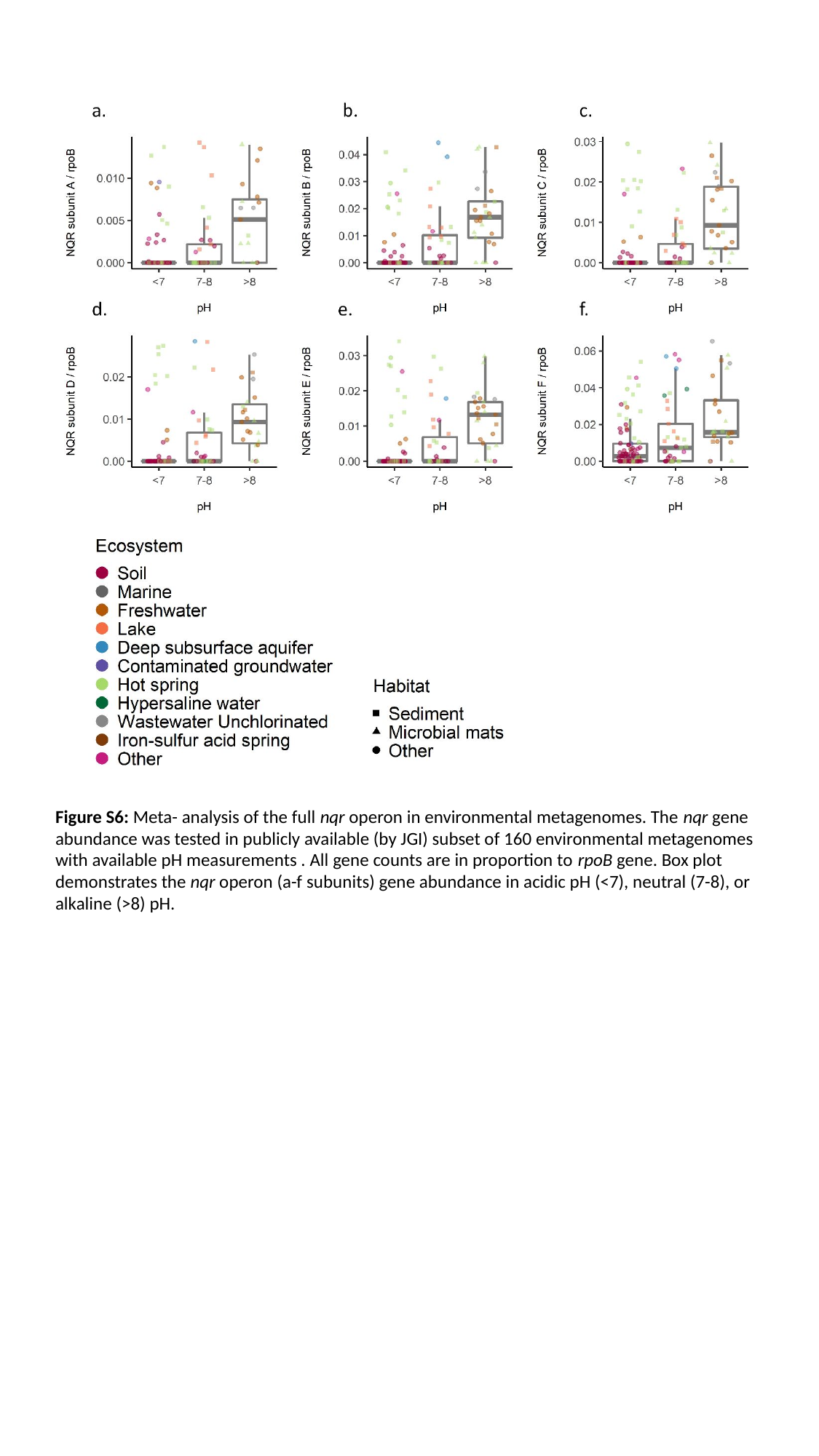

#
Figure S6: Meta- analysis of the full nqr operon in environmental metagenomes. The nqr gene abundance was tested in publicly available (by JGI) subset of 160 environmental metagenomes with available pH measurements . All gene counts are in proportion to rpoB gene. Box plot demonstrates the nqr operon (a-f subunits) gene abundance in acidic pH (<7), neutral (7-8), or alkaline (>8) pH.

## Slide 7
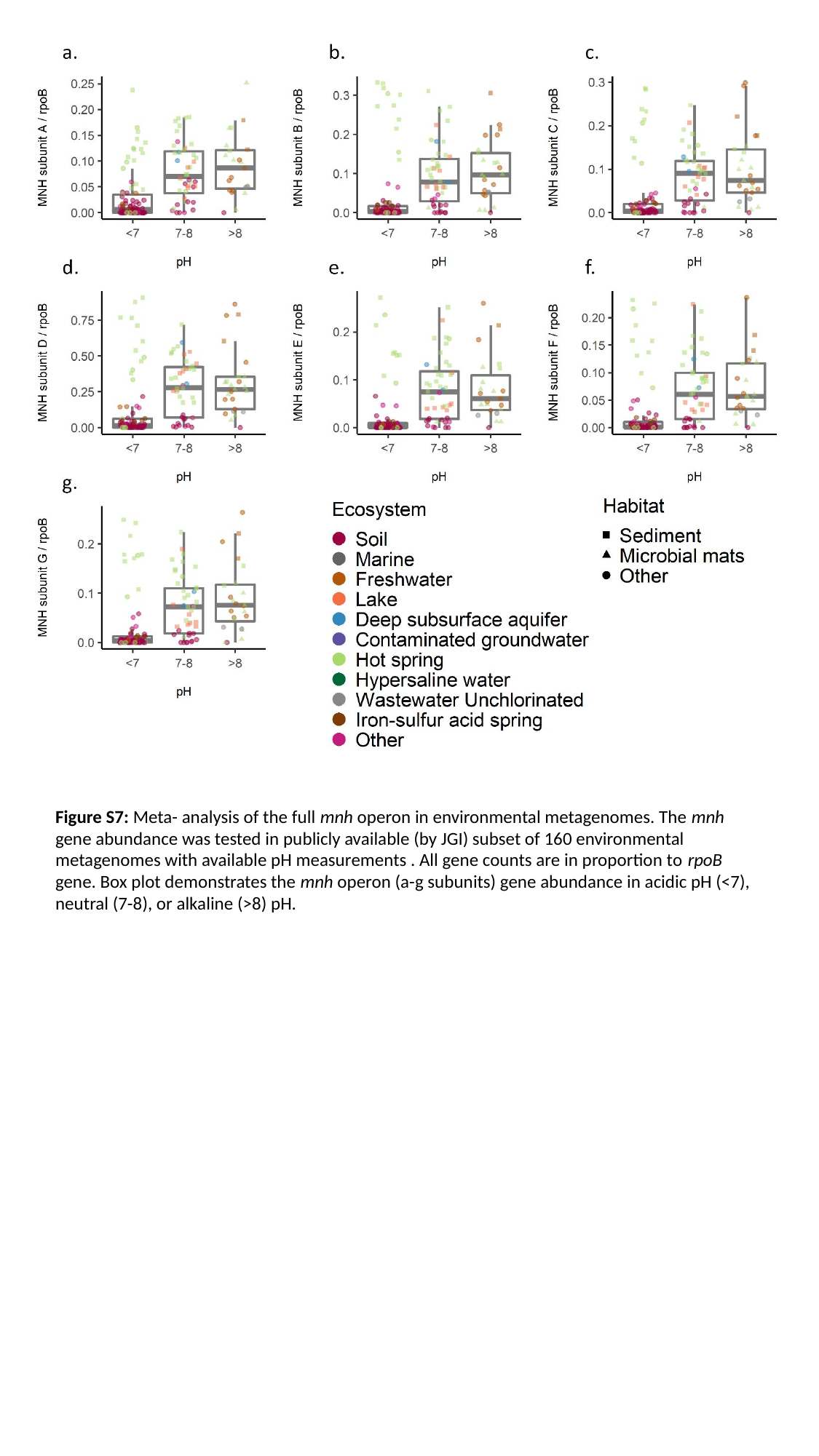

Figure S7: Meta- analysis of the full mnh operon in environmental metagenomes. The mnh gene abundance was tested in publicly available (by JGI) subset of 160 environmental metagenomes with available pH measurements . All gene counts are in proportion to rpoB gene. Box plot demonstrates the mnh operon (a-g subunits) gene abundance in acidic pH (<7), neutral (7-8), or alkaline (>8) pH.

## Slide 8
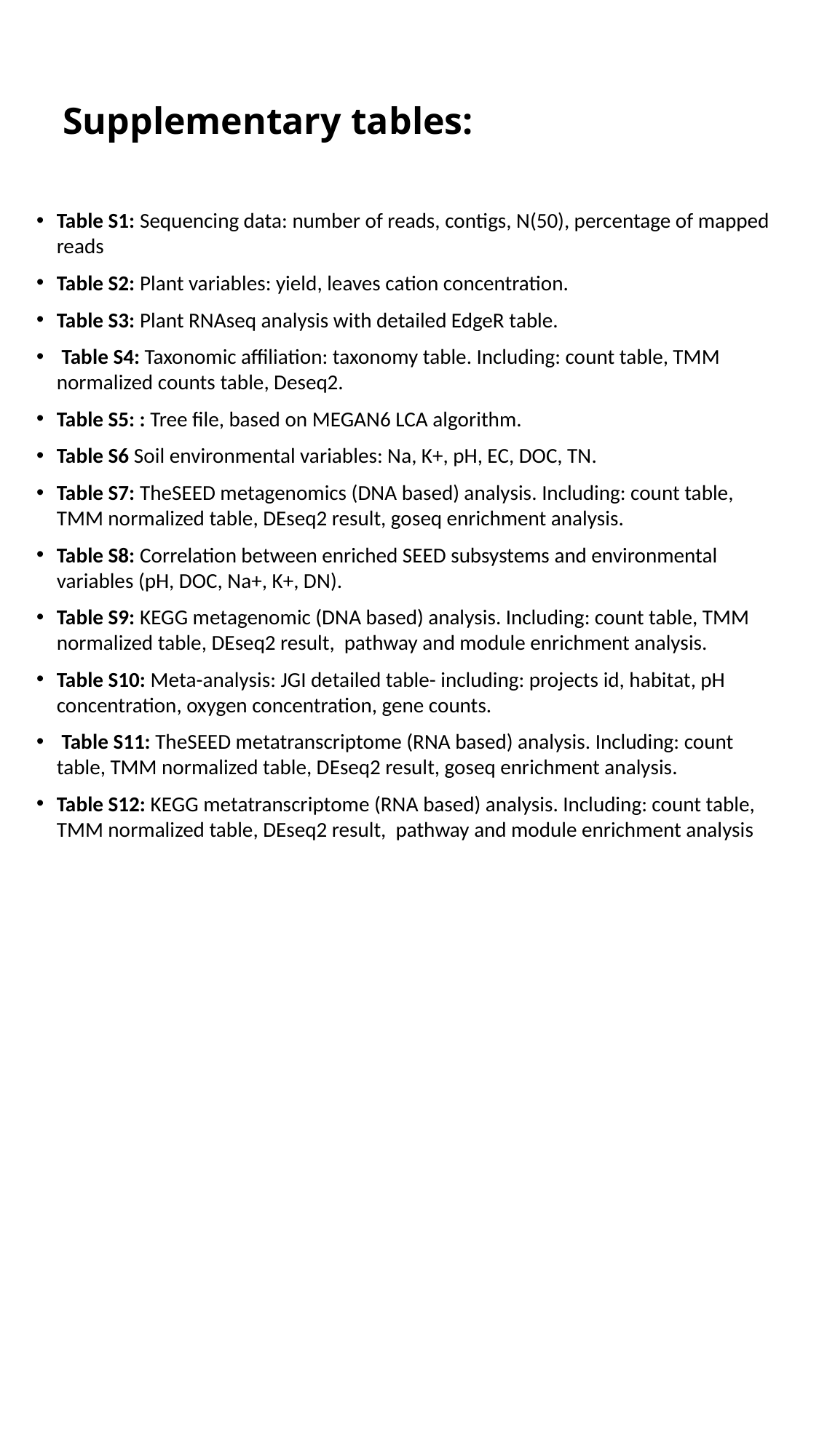

# Supplementary tables:
Table S1: Sequencing data: number of reads, contigs, N(50), percentage of mapped reads
Table S2: Plant variables: yield, leaves cation concentration.
Table S3: Plant RNAseq analysis with detailed EdgeR table.
 Table S4: Taxonomic affiliation: taxonomy table. Including: count table, TMM normalized counts table, Deseq2.
Table S5: : Tree file, based on MEGAN6 LCA algorithm.
Table S6 Soil environmental variables: Na, K+, pH, EC, DOC, TN.
Table S7: TheSEED metagenomics (DNA based) analysis. Including: count table, TMM normalized table, DEseq2 result, goseq enrichment analysis.
Table S8: Correlation between enriched SEED subsystems and environmental variables (pH, DOC, Na+, K+, DN).
Table S9: KEGG metagenomic (DNA based) analysis. Including: count table, TMM normalized table, DEseq2 result, pathway and module enrichment analysis.
Table S10: Meta-analysis: JGI detailed table- including: projects id, habitat, pH concentration, oxygen concentration, gene counts.
 Table S11: TheSEED metatranscriptome (RNA based) analysis. Including: count table, TMM normalized table, DEseq2 result, goseq enrichment analysis.
Table S12: KEGG metatranscriptome (RNA based) analysis. Including: count table, TMM normalized table, DEseq2 result, pathway and module enrichment analysis
